# Sulfamide chemistry applied to the functionalization of self-assembled monolayers on gold surfaces

**DOI:** 10.3762/bjoc.13.64

**Published:** 2017-04-04

**Authors:** Loïc Pantaine, Vincent Humblot, Vincent Coeffard, Anne Vallée

**Affiliations:** 1 Institut Lavoisier de Versailles, UMR 8180, Université Paris-Saclay, Université de Versailles Saint-Quentin, 45 avenue des Etats-Unis, 78035 Versailles Cedex, France; 2 Sorbonne Universités, UPMC Univ. Paris 06, Laboratoire de Réactivité de Surface, UMR CNRS 7197, 4 place Jussieu, 75005 Paris, France; 3 Université de Nantes, CNRS, CEISAM, UMR 6230, Faculté des Sciences et des Techniques, rue de la Houssinière, BP 92208, 44322 Nantes Cedex 3, France

**Keywords:** gold surfaces, hydrolysis, IRRAS, reversibility, SAM, sulfamide, XPS

## Abstract

Aniline-terminated self-assembled monolayers (SAMs) on gold surfaces have successfully reacted with ArSO_2_NHOSO_2_Ar (Ar = 4-MeC_6_H_4_ or 4-FC_6_H_4_) resulting in monolayers with sulfamide moieties and different end groups. Moreover, the sulfamide groups on the SAMs can be hydrolyzed showing the partial regeneration of the aniline surface. SAMs were characterized by water contact angle (WCA) measurements, Fourier-transform infrared reflection absorption spectroscopy (IRRAS) and X-ray photoelectron spectroscopy (XPS).

## Introduction

**Scheme 1:**
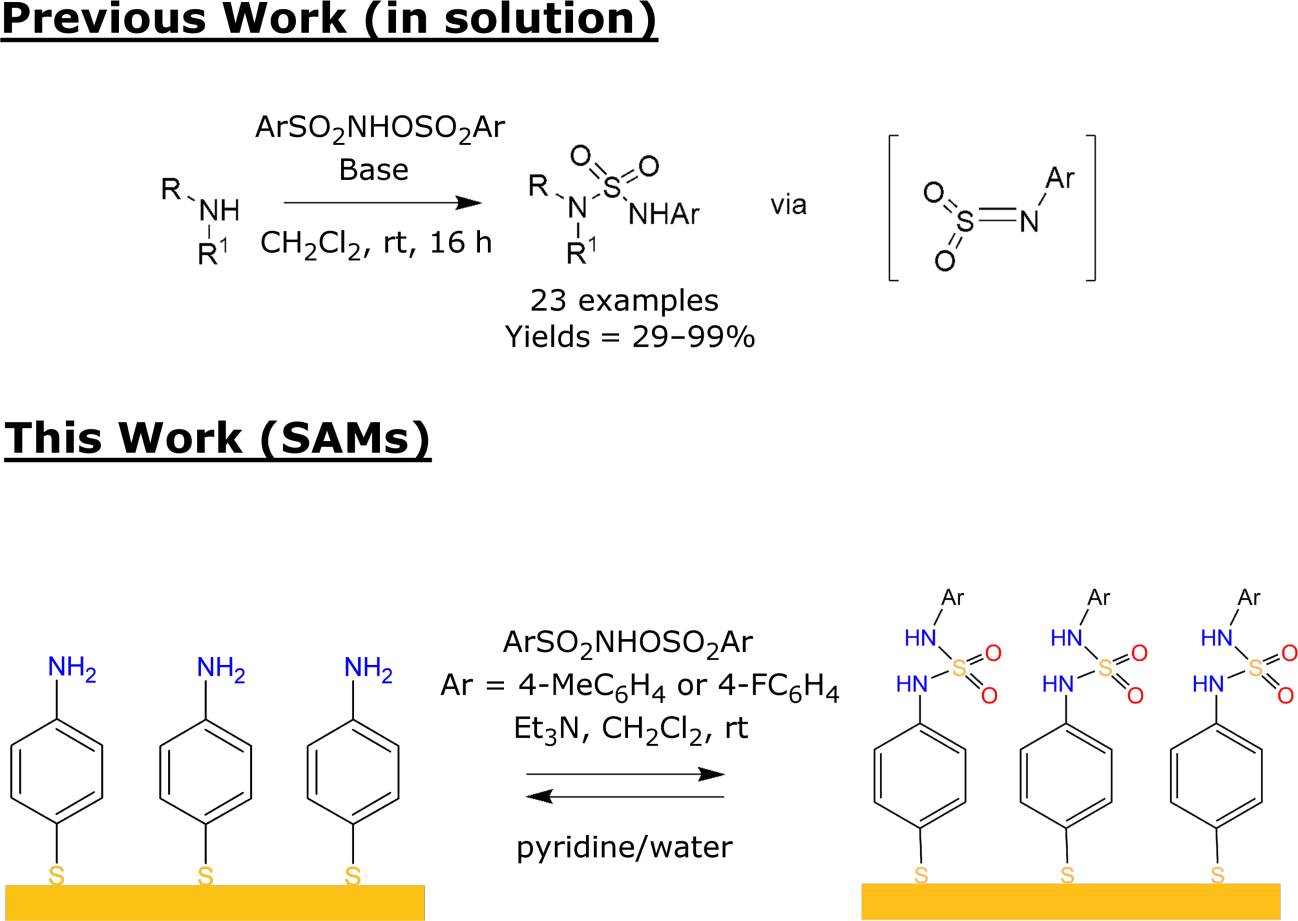
General strategy for surface functionalization based on sulfamide chemistry.

Self-assembled monolayers (SAMs) have raised considerable interest in the past decades because of their potential applications in various areas such as biomaterials, tissue engineering, biosensors and electronics [1–3]. The seminal work of Nuzzo and Allara on the adsorption of disulfides on gold surface has triggered numerous research activities in the preparation and applications of sulfur-based SAMs on Au surfaces [4]. Important contributions have been notably driven by the implementation of reactive end groups in the monolayers enabling the chemical functionalization of solid surfaces [3,5–7]. Within this context, noncovalent and covalent strategies have been investigated for the immobilization of a target molecule through a reaction with the terminal groups of the SAMs. The most common methods to covalently functionalize these materials involve the Huisgen cycloaddition between an azide and an alkyne [8–9], Thiol-Michael addition [10–11], amide formation [12–14], Diels–Alder reaction [15–16] or the imine/oxime condensation [17–18]. These reactions tend to produce strong covalent interactions between the surface and the molecules in solution which ensure a stable immobilization. One limitation of the covalent strategy lies in the irreversible permanent functionalization of the SAMs which precludes reusable properties. A reversible strategy could find applications in a wide range of fields such as the controlled engineering of SAMs, the formation of patterns with capture-and-release properties, the reusability of the surface for further functionalization or the ability to tune the properties of SAMs by controlled spatial functionalization. A scant number of examples have reported reversible covalent reactions on SAMs on gold surfaces; for instance, Ravoo and Reinhoudt have described the formation of imine SAMs prepared by reaction of an amino-terminated SAM with an aldehyde in solution or the condensation of an aldehyde-terminated SAM with an amine in solution [19]. These surfaces were stable in water but readily erased by acid-catalyzed hydrolysis at pH 3. The propensity of imines to be hydrolyzed under acidic conditions has also been harnessed for the formation of aromatic mixed self-assembled monolayers containing both imine functionalities and protonated anilines on the surface [20]. In order to bring a new class of reusable surfaces, we describe herein the use of sulfamide chemistry for the generation of reversible patterns of sulfur-based SAMs on a gold surface (Scheme 1). To the best of our knowledge, the formation of sulfamide for the chemical modification of monolayers on gold surfaces has never been reported.

The sulfamide functionality with R_2_NSO_2_NR’_2_ structure can be found in several biorelevant compounds [21]. Besides applications in medicinal chemistry, sulfamide groups have been incorporated in self-assembling molecules [22–27], peptides [28], polymers [29], ligands [30], chiral auxiliaries [31–33] and in organocatalysts [34–37]. In light of the importance of the sulfamide functionality, our group has recently reported a straightforward preparation of unsymmetrical sulfamides from commercially available amines and *N*-hydroxyarenesulfonamide *O*-derivatives under simple conditions [38–39]. The method works at room temperature without needing inert atmosphere or dry solvent. The ease of formation of sulfamides and their propensity to be cleaved under mild conditions [40] prompted us to consider the sulfamide functional group for the linkage and the potential regeneration of amine-terminated SAMs on gold surface.

Here, we present a new strategy to modify in situ amino terminated SAMs on gold based on the sulfamide chemistry and to partially regenerate the amino SAM. The surface modification process is studied by water contact angle measurements (WCA), Fourier transform infrared reflection absorption spectroscopy (PM-IRRAS) and X-ray photoelectron spectroscopy (XPS).

## Results and Discussion

In order to monitor the successful formation of the sulfamide functional group on a gold surface, a reference molecule sulfamide **1** was first synthesized to prepare a model sulfamide SAM (SAM 1). The XPS and infrared signatures of the sulfamide moiety obtained from sulfamide **1** SAM were systematically used as reference when analyzing the modified gold surfaces after the reaction process.

### Synthesis of sulfamide **1**

**Scheme 2:**
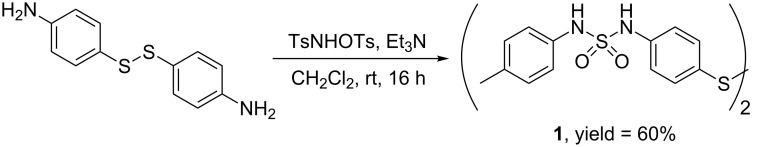
Synthesis of the reference molecule sulfamide **1**.

The disulfide **1** was synthesized following our previous procedure from commercially available 4-aminophenyl disulfide and the readily prepared 4-methyl-*N*-(tosyloxy)benzenesulfonamide in the presence of triethylamine (Scheme 2) [38]. The desired product sulfamide **1** was obtained in 60% yield after purification on silica gel and was fully characterized before the preparation of sulfamide-terminated SAMs (Supporting Information File 1).

### Sulfamide formation on gold substrates

**Figure 1:**
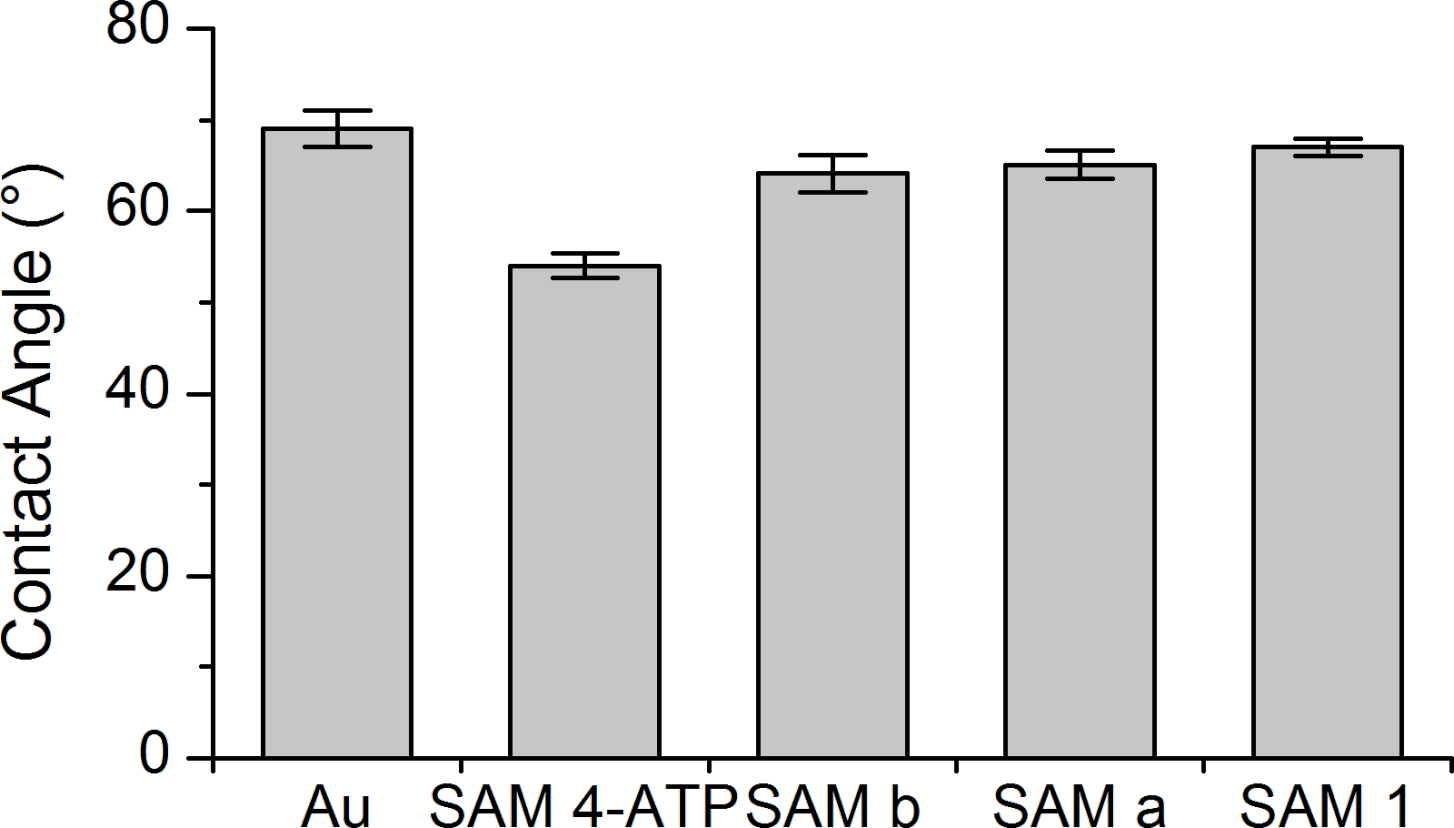
Contact angles of the gold surface, the 4-ATP SAM, the 4-ATP SAM after reaction with ArSO_2_NHOSO_2_Ar (Ar = 4-MeC_6_H_4_ or 4-FC_6_H_4_), respectively SAM a and SAM b and the sulfamide **1** SAM.

The elaboration of the aniline terminated surface (SAM 4-ATP) is the first step in creating SAMs bearing a sulfamide group, see Figure 1. For this purpose, 4-amino-thiophenol (4-ATP) was first adsorbed on gold surfaces. The aniline-terminated surface obtained can then react with ArSO_2_NHOSO_2_Ar (Ar = 4-MeC_6_H_4_ and 4-FC_6_H_4_), respectively SAM a and SAM b, to form sulfamide cross linkage. The reaction on the surface was investigated through contact angle measurements, PM-IRRAS and XPS analysis of the surfaces.

Water contact angle measurements were performed to investigate the hydrophilic character of grafted surfaces after the different reaction steps. The values presented in Figure 1 display water contact angles for bare Au around 67 ± 2° as expected for a clean gold surface [41]. Upon 4-ATP adsorption the water contact angle decreases compared to the clean gold sample with a value of 54 ± 2° indicating the increase of the hydrophilicity of the surface, which is in agreement with the formation of an amino-terminated monolayer [19].

SAM a (4-MeC_6_H_4_SO_2_NHOSO_2_-4-MeC_6_H_4_) and SAM b (4-FC_6_H_4_SO_2_NHOSO_2_-4-FC_6_H_4_) exhibit both a similar contact angle around 65 ± 2° showing a more hydrophobic nature of the SAMs after the reaction, which is coherent with the introduction of methyl or fluorine-terminated groups. The contact angles are lower than that for pure aromatic CF_3_ or CH_3_ terminated film ( ≈81° and ≈80°, respectively) [42]. This behaviour can be explained by two reasons. (1) The conversion of the coupling reaction is not complete, some amino groups still remain at the top of the layer and it contributes to the lower contact angles values observed. (2) The sulfamide moieties in the aromatic skeletons which are more hydrophilic than pure aromatic skeletons contribute to the decrease of the contact angle values compared to the pure aromatic layers. Moreover, as it was already observed in many works the F-containing SAM and the CH_3_ containing one, display similar contact angle values while F moiety is known to be more hydrophobic than the CH_3_ group [41–43]. As mentioned above, the SAMs a and b are probably heterogeneous, thus the hydrophobic end groups resulting of the coupling reaction are in the outer part of the monolayer and are free to become disordered [43]. Moreover, the F end group is smaller than the CH_3_ one and thus may be more flexible, inducing a more disordered layer and lowered hydrophobic properties than the one expected.

The reference SAM 1 which is not heterogeneous also exhibits a similar value, lower than the expected one. In this case, the lower value can be explained by the sulfamide moieties in the aromatic skeletons which are more hydrophilic than pure aromatic skeletons but also by a more disordered layer as it is common for SAM prepared with big molecules.

Although the water contact angle measurements suggest the formation of the sulfamide moieties, in this work the contact angle values are very similar for the SAMs a, b, **1** and the gold bare. This technique is therefore not sufficient alone to ascertain the good formation of the conversion; the different samples have also been characterized by PM-IRRAS and XPS.

**Figure 2:**
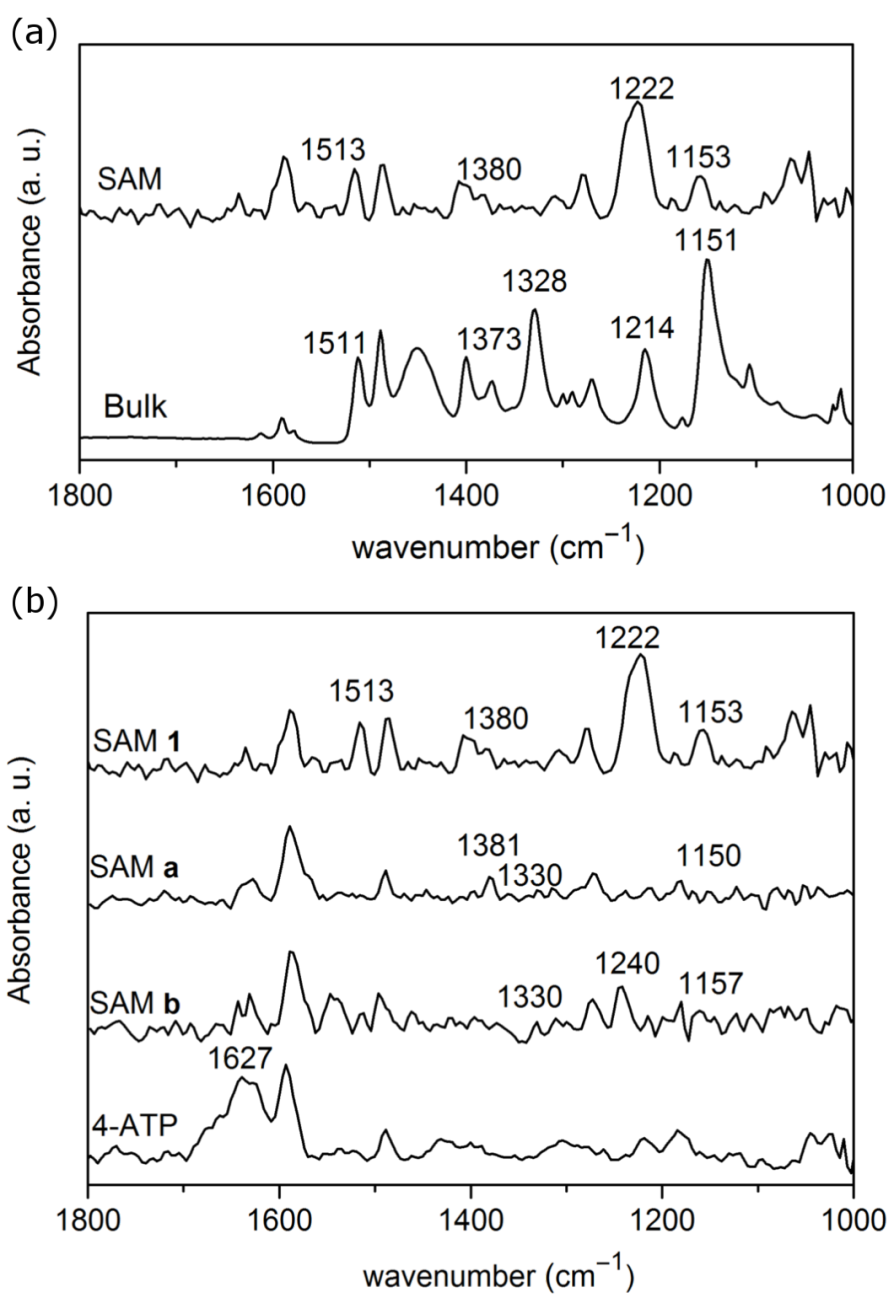
(a) IR spectra of sulfamide **1** in bulk (solid state) (bottom) and adsorbed on gold (top). (b) PM-IRRAS spectra of the 4-ATP SAM. The 4-ATP SAM is reacted with 4-FC_6_H_4_SO_2_NHOSO_2_-4-FC_6_H_4_ (SAM b) or 4-MeC_6_H_4_SO_2_NHOSO_2_-4-MeC_6_H_4_ (SAM a) and the sulfamide **1** SAM (SAM 1).

The PM-IRRAS spectrum of the sulfamide **1** SAM and the ATR spectrum of sulfamide **1** at solid state are shown in Figure 2a. Detailed bands assignments are summarized in Table 1. The general spectroscopic profiles in two different states are comparable, which suggests the successful adsorption of the sulfamide **1** on the gold surface. There is quite a good agreement between both spectra, and the differences observed can be explained by the specificity of both IR techniques; while ATR will provide information from the bulk; in opposite, IRRAS following the metal surface selection rules (MSSR [44]) implies that only dipoles perpendicular to the surface will be observed. The in-plane aromatic C=C vibrational modes and in-plane C–H deformation are summarized in Table 1, but the presence of two benzene rings on the sulfamide makes the interpretation of the molecule orientation on the surface difficult. Additionally to these vibrations, the two spectra show a band at ≈1380 cm^−1^ attributed to the symmetric deformation of the terminal methyl group [45] of the sulfamide **1** and two bands at ≈1220 and ≈1511 cm^−1^ which is assigned to C–N stretching mode and the N–H deformation of the sulfamide bond, respectively. On the bulk spectrum, the symmetric and asymmetric SO_2_ stretching modes are identified at 1328 and 1151 cm^−1^, respectively [46]; while the single asymmetric SO_2_ vibration is observed on the SAM PM-IRRAS spectra at 1153 cm^−1^. Therefore, by application of the strict IRRAS dipole selection rules, the SO_2_ group should be oriented parallel to the surface.

**Table 1:** Assignment of the vibrational modes probed by PM-IRRAS.

Assignement	Sulfamide **1**		SAM a	SAM b	SAM 4-ATP

	Bulk	SAM			

δ^NH^ primary amine			1627	1627	1627
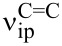 ring (a1)	1591	1589	1590	1590	1592
δ^NH^ moiety	1511	1513		1511 (w)	
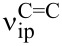 ring (a1)	1488	1484	1488	1488	1488
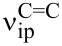 ring (b2)	1450				
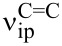 ring (b2)	1400				
δ_CH3_	1373	1380	1381		
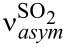	1328		1330 (w)	1330 (w)	
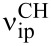 ring (b2)	1268	1276	1275	1275	1261
ν^CF^ring				1240	
ν^CN^	1214	1222	1214 (w)		1218
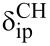 ring (a1)	1179	1184	1180	1180	1179
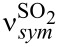	1151	1153	1150 (w)	1157 (w)	
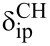 ring (b2)		1122 (vw)	1627	1627	1122
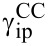 ring (a1)	1106				

The PM-IRRAS spectrum of 4-ATP on gold is shown in Figure 2b and is dominated by a band at 1627 cm^−1^ assigned to deformation modes of the amino group and bands of the benzene skeleton with a_1_ symmetries at 1592, 1488 cm^−1^ and the in plane CH bending at 1179 cm^−1^, as it was already observed in the literature [47]. Other weak bands, at 1261 and 1122 cm^−1^ are also visible on the spectrum and are attributed to the in plane CH deformations with b_2_ symmetry of the benzene skeleton. Again, according to the IR metal surface selection rules, the lower relative ratio intensities of the b_2_ vibration modes compared to the a_1_ vibrations in the SAM compared to the one of the 4-ATP bulk (Supporting Information File 1, Figure S2), suggests that the 4-ATP benzene ring is oriented perpendicular to the surface with a small tilt angle to the surface normal.

After exposure of the 4-ATP SAM to TsNHOTs, several changes are observed in the spectrum; previous bands observed in the 4-ATP SAM spectrum are still present with several new bands appearing due to SAM b. The CH_3_ deformation vibration at 1381 cm^−1^ and two weak bands at 1150 and 1330 cm^−1^ are assigned respectively to the following vibrational modes 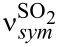
and 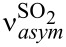. The presence of these characteristic methyl and sulfamide bands already observed in the sulfamide **1** IR spectrum provides evidence that a part of the 4-ATP molecules have reacted with TsNHOTs. The absence of the δ_NH_ sulfamide moiety and the appearance of the 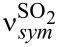 compared to the sulfamide **1** SAM spectra can be explained by difference on the molecule orientation on the surface. To show the possibility to extend the reaction, the same experiment was performed with substituted 4-FC_6_H_4_SO_2_NHOSO_2_-4-FC_6_H_4_. The SAM b spectrum is very similar to the one of SAM a, Figure 2b. The main differences are the absence of the methyl deformation and the presence of a band at 1240 cm^−1^ assigned to C–F stretching mode of fluorobenzene moiety [48].

XPS experiments were also performed to analyze the modified surfaces, and the data confirmed the formation of the sulfamide groups (mainly with the appearance of the SO_2_ spectroscopic signature at high binding energy around 168 eV). The conversion rate of the reaction has also been calculated with elemental atomic analysis

Carbon, nitrogen, sulfur, oxygen and gold were observed on the different surfaces by XPS spectra and an additional fluor F1s contribution was also observed on the surface of SAM b (4-FC_6_H_4_SO_2_NHOSO_2_-4-FC_6_H_4_).

**Figure 3:**
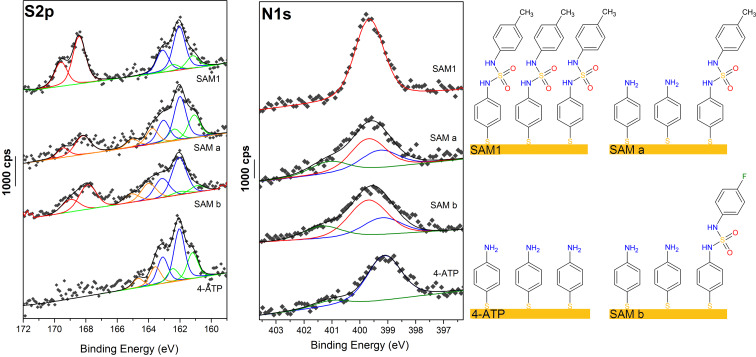
High resolution S2p and N1s XPS spectra of the 4-ATP SAM, the 4-ATP SAM after reaction with 4-FC_6_H_4_SO_2_NHOSO_2_-4-FC_6_H_4_ (SAM b) or TsNHOTs (SAM a) and the sulfamide 1 SAM (SAM 1). Right panel: Schematic view of the different SAMs created on gold surfaces.

High resolution N1s and S2p XPS signals are presented in Figure 3. Before the reaction leading to the SAMs a or b, the 4-ATP SAM N1s peak presents two contributions at 399.2 and 401.2 ± 0.1 eV, respectively, attributed to nitrogen of deprotonated (≈91%) and protonated (≈9%) amine groups [49]. The SAM 1 surface N1s peak highlights only one thin contribution at 399.7 ± 0.1 eV attributed to sulfamide nitrogen (-NH-SO_2_-NH-); notably, the N1s peaks of the SAMs a and b are best fitted with three contributions at 399.2 eV, 399.7 eV and 401.2 eV ± 0.1 eV corresponding to a mixture of 4-ATP and molecules with sulfamide groups on the surface confirming that the reaction occurs as it was previously observed by PM-IRRAS and contact angle measurements.

In this work the S2p signal is particularly important because it allows the characterization of the SAM formation via thiol moieties and especially evaluating the conversion rate of the reaction since the XPS signature of the sulfamide moiety must be very different from the one of thiol moiety.

The all four samples highlight a strong S2p_3/2_,_1/2_ doublet at 162.0 ± 0.1 eV (S2p_3/2_) (blue) characteristic of the thiolate-gold bond [50] with an additional XPS peaks doublet at lower binding energy of 161.1 ± 0.1 eV (green) attributed to multicoordinated sulfur bond to the gold surface [51]. On the 4-ATP, a and b SAMs, a minor S2p signal at 163.6 eV (orange) is allocated to free thiol suggesting that a small fraction of thiol groups (≈16–19%) are not bonding via the sulfur atom. This contribution is not observed in the SAM 1 showing no unbound molecules in the SAM. It can be explained by the different preparation procedure.

It is known that oxidised sulfur highlights a doublet at high binding energy 167–169 eV but since there was no XPS data to our knowledge in the literature on sulfamide, the analysis of the reference SAM 1 is crucial. The S2p spectrum of the reference sulfamide **1** SAM is dominated by a strong doublet with S2p_3/2_ peak at 168.4 ± 0.1 eV (red) assigned to sulfamide moiety (≈47% of total sulfur intensity). This latter attribution is confirmed by the relative intensity ratio characteristics of the molecule S_sulfamide_/(S_bound_ + S_unbound_) equal to 0.9 and N/S_sulfamide_ equal to 2.2, which are very close to the theorical expected values of 1 and 2 respectively. This contribution at 168.4 ± 0.1 eV assigned to the sulfamide moiety is clearly present on the SAMs a and b S2p signal showing the formation of a sulfamide moiety.

The SAM b surface XPS analysis of the F1s region show one symmetric peak at 687.3 ± 0.1 eV suggests a single fluorine environment on the surface, which is assigned to the fluorobenzene group (Supporting Information File 1, Figure S3) [52].

The conversion rate can be evaluated by comparing the area of the sulfamide contributions with the area of the bounded and unbounded sulfur on the S2p signal. The conversion rate with SAM a (TsNHOTs) and SAM b (4-FC_6_H_4_SO_2_NHOSO_2_-4-FC_6_H_4_) was estimated to be 31 and 47%, respectively. This conversion rates can be cross-checked by comparing the area of the sulfamide contributions with the area of protonated and not protonated amino groups on the N1s signal. The conversion rate obtained by this way is very similar to the one obtained from the S2p signal, 32 and 48% for the SAM a and SAM b, respectively.

All characteristic ratios of the SAMs a and b obtained by XPS have been compared with the theoretical ratios calculated from the conversion rate estimated (Table 2). The good agreements between the values confirm that the reaction occurs.

**Table 2:** Experimental (XPS) and theoretical characteristic ratios of the 4-ATP SAM, the 4-ATP SAM after reaction with 4-FC_6_H_4_SO_2_NHOSO_2_-4-FC_6_H_4_ (SAM b) or TsNHOTs (SAM a) and SAM 1. The theoretical ratios were calculated with a conversion rate of 47 and 31% for SAM b and SAM a, respectively.

	N/S	S_S=O_/(S_bound_+ S_unbound)_	F/S	N/S_S=O_	N/(S_bound_+ S_unbound)_	N_sulf_/ (N_NH2_+ N_NH3+)_	N_sulf_ /S_S=O_

4-ATP (XPS)	0.96	–	–	–	0.96	–	–
4-ATP (theorical)	1	–	–	–	1	–	–

SAM a (XPS)	1	0.31	–	4.28	1.30	1.00	1.30
SAM a (theorical)	1	0.31	–	4.27	1.31	0.90	1.31

SAM b (XPS)	1.2	0.47	0.32	3.90	1.84	1.84	2,53
SAM b (theorical)	1	0.47	0.32	3.13	1.47	1.77	2

SAM 1 (XPS)	1	0.89	–	2.25	2	–	2
SAM 1 (theorical)	1	1	–	2	2	–	2

### Sulfamide hydrolysis

As previously mentioned, reversible covalent chemistry on surfaces opens many potential applications but it is very little developed on gold surfaces. One of the main reasons could be explained by the necessity to work under mild conditions; it is well known that the energy of interaction between sulfur and gold is in order of 45–50 kcal/mol and the desorption of the thiols can occur at about 70 °C in hydrocarbon solvent [53].

The work of Crampton showed the possibility to cleave the sulfamide group under mild conditions in solution to obtain the corresponding free amines [40]. In order to explore the possibility to cleave the sulfamide linkage on the surface to obtain the aniline terminated SAM surface, it is essential first to determine the best reaction conditions. The conversion rate of the hydrolysis of model sulfamide molecule in solution at four different temperatures 40, 60, 70 and 80 °C was first investigated by ^1^H NMR. The results are shown in Supporting Information File 1, Figure S4.

To ensure the integrity of the SAM layer on gold, a temperature of 70 °C for the hydrolysis of the SAM 1 was chosen. It corresponds to a reaction yield about 65% in solution after two hours of reaction.

The sulfamide hydrolysis on the SAM surface is tested towards SAM 1 to show the possibility to recover the initial surface**,** after treatment with a mixture 5% H_2_O-pyridine at 70 °C after two hours.

**Figure 4:**
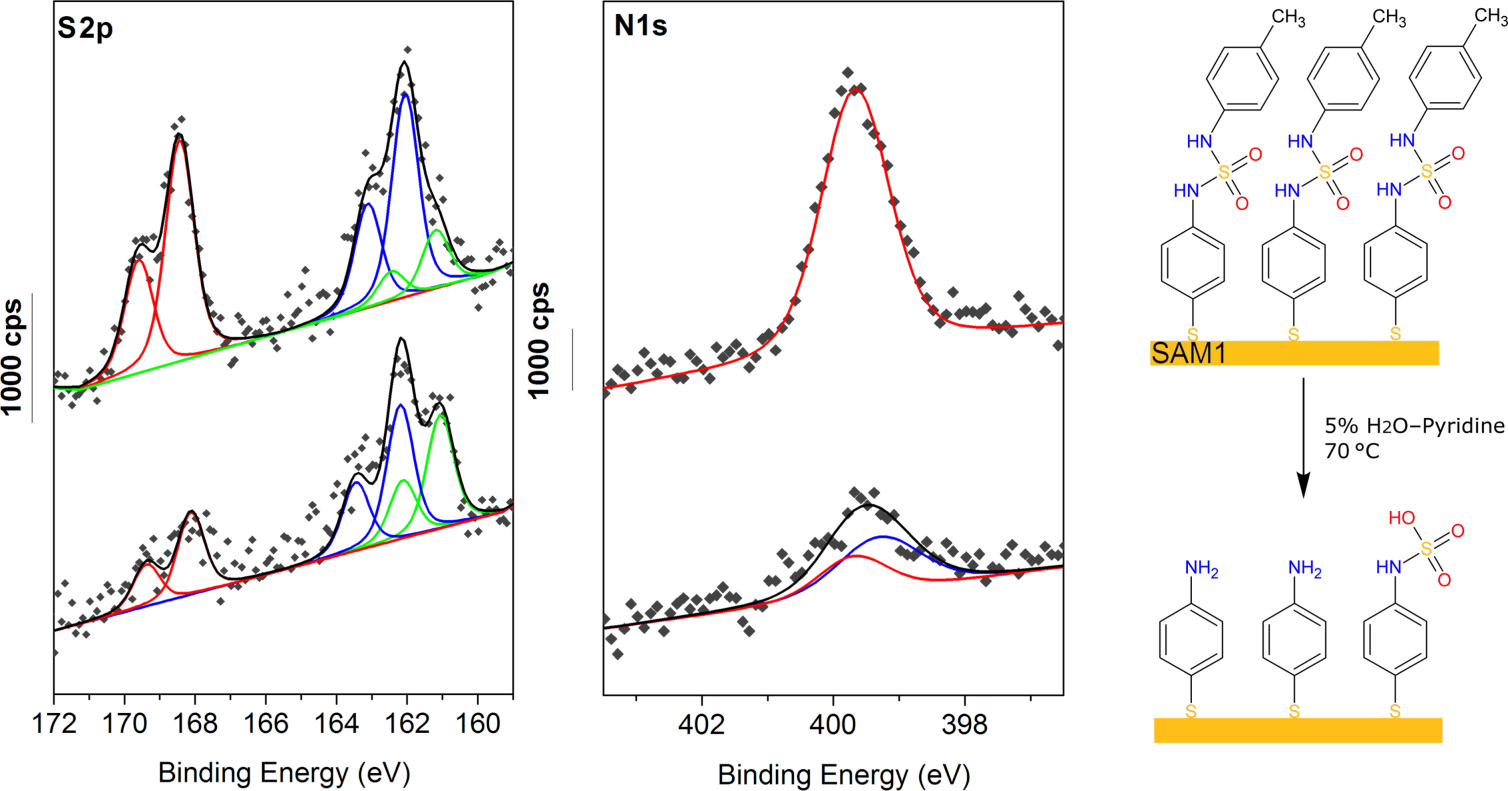
High resolution S2p and N1s XPS spectra of the SAM 1 before (top) and after hydrolysis (bottom). Right panel: Schematic view of the SAMs hydrolysis.

XPS spectra show that carbon, nitrogen, sulfur, oxygen and gold are still present on the surface. High resolution N1s and S2p XPS signals of the surface before and after hydrolysis are very different (Figure 4). There is a decrease of both S2p and N1s signal intensity due to the sulfamide cleavage. The characteristic ratios are shown in the Table 3. The decrease of the N_total_/S_bounded_ ratio from 1.99 to 0.87 and the increase of N_total_/S_S=O_ from 2.2 to 2.7 after the hydrolysis highlights that sulfamide **1** is cleaved leading to the formation of the initial aniline. However, the presence of contribution at high binding energy around 168.4 eV assigned to oxidized sulfur (≈33%) in the S2p high resolution XPS spectra shows that the hydrolyse is not quantitative. The decrease of the N_total_/S_total_ ratio, suggests the formation of additional sulfamic acid derived from 4-ATP. As a matter of fact, the sulfamic acid moiety has a contribution at 168.4 eV in the S2p signal, the same binding energy than the sulfamide moiety. This can be explained by the fact that the sulfur in the sulfamic acid moiety is surrounded with three oxygens and one nitrogen (-NH-SO_3_H-) and the sulfur in the sulfamide moiety is surrounded with two oxygens but two nitrogens (-NH-SO_2_-NH-). In total the two kinds of sulfur are surrounded with four heteroatoms leading to a contribution at the same binding energy for the two sulfur atoms. Moreover, the N1s peak of the surface after hydrolysis can be fitted with two contributions at 399.3 and 399.7 ± 0.1 eV, attributed to amino group (≈66%) and sulfamic acid moiety (34%), respectively. One can note the absence of protonated amino group which should be observed around 401–402 eV. This can be easily explained by the use of pyridine, a strong base, during the hydrolysis process. Additionally, the low energy contributions attributed to multicoordinated sulfur bounds to the gold surface in the S2p signal increased after the hydrolysis. This phenomenon may be due to the heating process during the hydrolysis. However, to be sure that no thiols were desorbed during the hydrolysis, the S_bound_/Au4f signal ratio before and after the hydrolysis is compared and is quite similar (e.g. 0,034 and 0.032, respectively). We concluded that the hydrolysis process did not induce any desorption of thiols but may have modified the layer organization.

Even if the reaction is not completely reversible, it is worth noting that the conversion rate of the hydrolysis in these mild conditions on the surface is as good as the one obtained in solution in the same conditions, e.g. 65%. While most of the previous works used only contact angle measurements to prove the reversibility of their process, a careful characterization of the surface has been carried out in this study [19].

**Table 3:** Experimental (XPS) and theoretical characteristic ratios of the SAM 1 after hydrolysis. The theoretical ratios were calculated with a surface showing 67% of 4-ATP and 33% of the sulfamic acid derived from 4-ATP.

	N/S	S_S=O_/(S_bound_+ S_unbound)_	N/S_S=O_	N/(S_bound_+ S_unbound)_

SAM 1 after hydrolysis (XPS)	0.65	0.33	2.62	0.87
SAM 1 after hydrolysis (theorical)	0.75	0.33	3.02	1

## Conclusion

In conclusion, a new reaction on gold surfaces was reported based on sulfamide chemistry. In this work, two sulfamide species with different functional end groups have been prepared in 31% and 47% conversions from readily available aniline-terminated self-assembled monolayers. The resulting sulfamide-derived SAMs were characterized by water contact measurements, Fourier-transform infrared reflection absorption spectroscopy and X-ray photoelectron spectroscopy. In addition, hydrolysis studies have been carried out both in solution and with sulfamide-derived SAMs. Under relatively mild conditions, the partial regeneration of the 4-ATP surface has been observed by hydrolysis of a sulfamide-derived SAM. This strategy paves the way to future applications in materials science and the results will be reported in due course.

## Experimental

### Synthesis of sulfamide **1**

Triethylamine (0.75 mmol, 105 µL, 3 equiv) was added to a solution of bis(4-aminophenyl) disulfide (0.25 mmol, 62 mg, 1 equiv) in dichloromethane (1 mL). The solution was cooled down to approximately 10 °C. ArSO_2_NHOSO_2_Ar (Ar = 4-MeC_6_H_4_) (0.55 mmol, 188 mg, 2.2 equiv) was dissolved in dichloromethane (1 mL) and added dropwise to the cooled solution. The reaction was then left to warm up to room temperature for 16 h. Water (2 mL) was added to the solution. The phases were separated and the aqueous phase was extracted with dichloromethane. The organic phases were combined, dried on anhydrous magnesium sulfate, filtered and concentrated under reduced pressure. The crude product was then purified by preparative chromatography on silica gel (pentane/EtOAc, 1/1) to afford the desired sulfamide **1** as a white solid (88 mg, 60% yield). mp: 183–186 °C; ^1^H NMR (300 MHz, DMSO-*d*_6_, 20 °C) δ 2.19 (s, 6H), 6.97 (app. d, *J*(H,H) = 8.4 Hz, 4H), 7.04 (app. d, *J*(H,H) = 8.4 Hz, 4H), 7.10 (app. d, *J*(H,H) = 8.7 Hz, 4H), 7.37 (app. d, *J*(H,H) = 8.7 Hz, 4H), 10.10 (s, 2H), 10.32 (s, 2H); ^13^C NMR (75 MHz, DMSO-*d*_6_, 20 °C) δ 20.2 (2C), 118.6 (4C), 119.1 (4C), 129.2 (2C), 129.4 (4C), 130.5 (4C), 132.3 (2C), 135.2 (2C), 138.6 (2C); IR (neat): 3291, 3031, 2918, 2857, 1449, 1329, 1150, 903, 807, 622, cm^-1^; HRMS–ESI (*m*/*z*): [M + H]^+^ calcd for C_26_H_27_N_4_O_4_S_4_ 587.0915, found: 587.0920.

### Monolayer preparation

A solution of 4 aminothiophenol (4-ATP, Fluka Inc. ≥95%) was prepared at 0.001 M in absolute ethanol.

The gold surfaces are constituted of glass substrates (11 mm × 11 mm), successively coated with a 50 Å thick layer of chromium and a 200 nm thick layer of gold, were purchased from Arrandee (Werther, Germany). The gold-coated substrates were annealed in a butane flame to ensure a good crystallinity of the topmost layers and rinsed in a bath of absolute ethanol during 15 min before adsorption.

All SAM preparations hve been performed on cleaned gold samples checked by polarisation modulation reflection absorption infrared spectroscopy (PM-IRRAS) and water contact angle (WCA) analysis.

Gold samples were modified with 4-ATP by 24 h of immersion in 0.001 M solutions in absolute ethanol, and rinsed successively with absolute ethanol (10 min), Milli-Q water (5 min), absolute ethanol (5 min) and dried under nitrogen flow.

Gold samples were modified with sulfamide **1** by 24 h of immersion in 0.001 M solutions in dichloromethane, and rinsed with successive bath of dichloromethane, absolute ethanol, Milli-Q water, and absolute ethanol during 5 min each and dried under nitrogen flow.

### Sulfamide formation on gold substrates

The gold surface functionalized by 4-ATP was immersed in 5 mL of dichloromethane; triethylamine (110 µL) was added to the stirring solution. The solution was cooled down to approximately 10 °C. ArSO_2_NHOSO_2_Ar (Ar = 4-MeC_6_H_4_, SAM a) or (Ar = 4-FC_6_H_4_, SAM b) (0.6 mmol) was dissolved in 1 mL and dichloromethane and added dropwise to the cooled solution. The reaction was then left to warm up to room temperature for 4 h. The gold surface was removed, rinsed successively in absolute ethanol (5 min), dichloromethane (5 min), Milli-Q water (5 min) and finally in absolute ethanol (5 min).

### Hydrolysis of model sulfamide molecule: NMR studies

10 mg of a *para-*toluene-derived sulfamide (4-MeC_6_H_4_NHSO_2_NH-4-MeC_6_H_4_) are dissolved in 0.5 mL of pyridine-*d*_5_. 50 μL of D_2_O is added to the mixture and the reaction mixture is placed in an NMR tube and analyzed with a 300 MHz spectrometer at different temperatures to determine the proportion of para-toluidine formed during the hydrolysis as a function of the imposed temperature (40, 60, 70 and 80 °C).

### In situ hydrolysis of sulfamide **1** SAM on gold

The hydrolysis of sulfamide compounds was carried out by immersion of gold SAM 1 presenting sulfamide **1** monolayer in 5% H_2_O–pyridine (5 mL) at 343 K for two hours under stirring. After the reaction, the samples were rinsed in successive baths of absolute ethanol, Milli-Q water and absolute ethanol during 10 min each and dried under nitrogen flow.

The surfaces were analysed by water contact angle, PM-IRRAS and X-ray photoemission spectroscopy (XPS).

PM-IRRAS analyses were performed in the air with the crystal placed in the external beam of a Fourier transform infrared Nicolet 5700 spectrometer. The experimental setup was described in a previous paper [50]. All reported spectra are recorded at 8 cm^−1^ resolution by co-addition of 128 scans; using the modulation of polarization techniques enabled us to perform rapid analyses of the samples after immersion without purging the atmosphere or requiring a reference spectrum.

XPS analyses were collected on Thermo Scientific ESCALAB 250 Xi and Omicron Argus X-ray photoelectron spectrometers. The X-ray source was Al Kα radiation (1486.6 eV) monochromatized radiation with a pass energy of 20 eV. The emissions of photoelectrons from the sample were analyzed at a take-off angle of 90° under UHV conditions. After collection, the binding energies (BE) were calibrated against the Au4f_7/2_ BE at 84.0 eV. The accuracy of the reported binding energies can be estimated to be ± 0.1 eV. The XPS peak areas were determined after subtraction of a background. Element peak intensities were corrected by Scofield factors [54]. All spectrum processing was carried out using Thermo Scientific™ Avantage Data System software or Casa XPS v.2.3.15 (Casa Software Ldt., UK). The spectral decomposition was performed by using Gaussian–Lorentzian (70%/30%) functions.

### Water contact angle measurements

Static water contact angles were measured under ambient conditions (at 20 °C and 40% relative humidity) analyzing the drop profile of sessile drops. 1 µL droplet of Milli-Q water was deposited on the sample surface using a Krüss DSA100 apparatus (Germany) equipped with a CCD camera and an image analysis processor. 4 droplets were analyzed on different locations on each sample and the test was performed in triplicate. The reported values are the averages of these 12 measurements for each kind of surface.

## Supporting Information

File 1 Sulfamide **1** NMR, IR spectra of 4-ATP in bulk and adsorbed on gold, F1s XPS spectrum of SAM b and hydrolysis diagram of *para-*toluene-derived sulfamide.
