# Supplementary material for: Sulfamide chemistry applied to the functionalization of self-assembled monolayers on gold surfaces
Source: Beilstein J Org Chem. 2017 Apr 4;13:648–58. doi: 10.3762/bjoc.13.64 (PMC5389194; doi:10.3762/bjoc.13.64)
Supplement: File 1 — Sulfamide 1 NMR, IR spectra of 4-ATP in bulk and adsorbed on gold, F1s XPS spectrum of SAM b and hydrolysis diagram of para-toluene-derived sulfamide. [file Beilstein_J_Org_Chem-13-648-s001.pdf]

Supporting Information

for

Sulfamide chemistry applied to the functionalization of self-assembled monolayers on gold surfaces

Loïc Pantaine[1], Vincent Humblot[2], Vincent Coeffard*[,3] and Anne Vallée*[,1]

Address: [1]Institut Lavoisier de Versailles, UMR 8180, Université Paris-Saclay, Université de Versailles Saint-Quentin, 45 avenue des Etats-Unis, 78035 Versailles Cedex, France, [2]Sorbonne Universités, UPMC Univ. Paris 06, Laboratoire de Réactivité de Surface, UMR CNRS 7197, 4 place Jussieu, 75005 Paris, France and [3]Université de Nantes, CNRS, CEISAM, UMR 6230, Faculté des Sciences et des Techniques, 2 rue de la Houssinière, BP 92208, 44322 Nantes Cedex 3, France

Email: Anne Vallée - anne.vallee@uvsq.fr; Vincent Coeffard - vincent.coeffard@univ-nantes.fr

*Corresponding author

Sulfamide 1 NMR, IR spectra of 4-ATP in bulk and adsorbed on gold, F1s XPS spectrum of SAM b and hydrolysis diagram of *para*-toluene-derived sulfamide

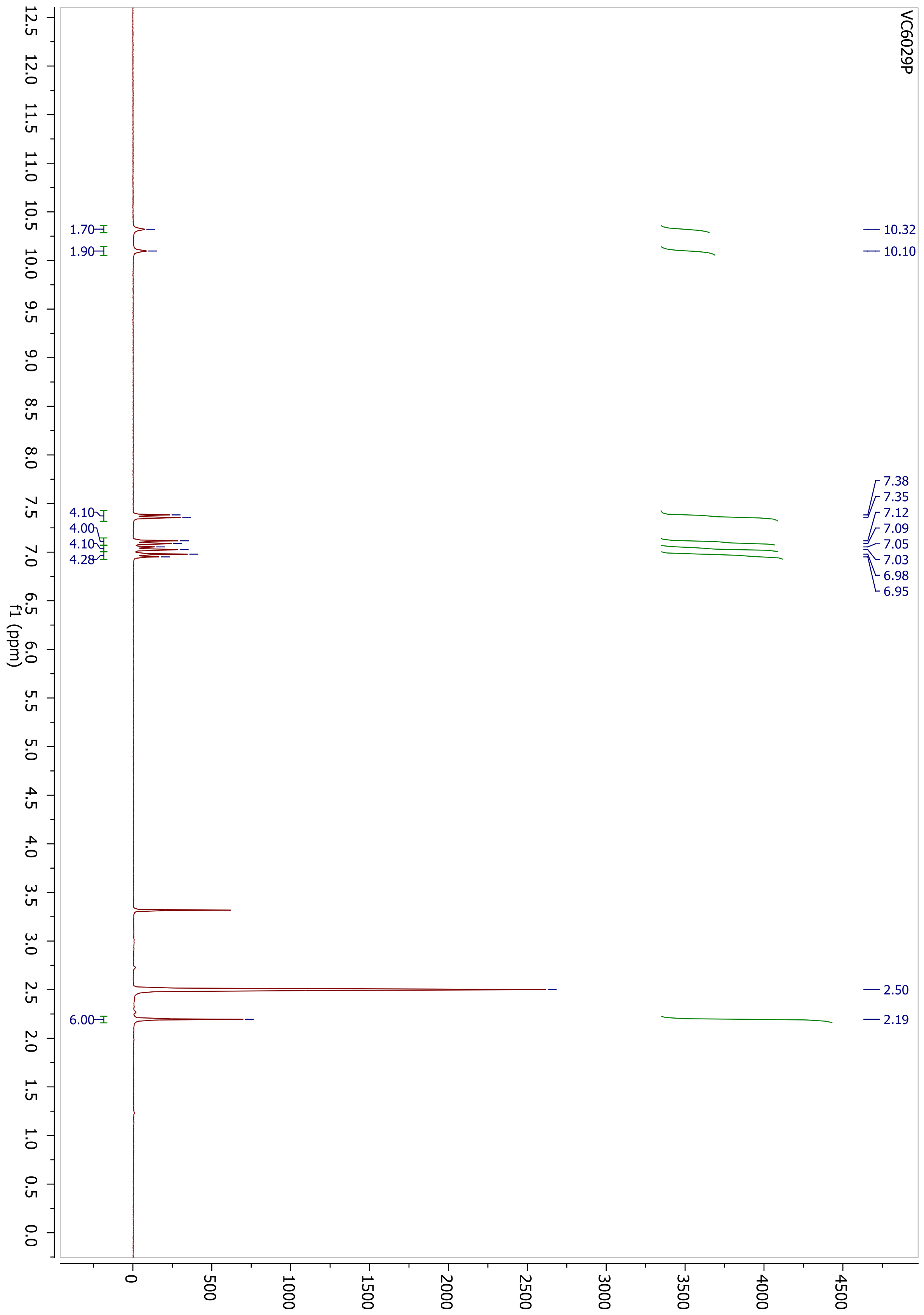
VC6029P
f1 (ppm)
10.32
10.10
7.38
7.35
7.12
7.09
7.05
7.03
6.98
6.95
2.50
2.19
1.70
1.90
4.10
4.00
4.10
4.28
6.00

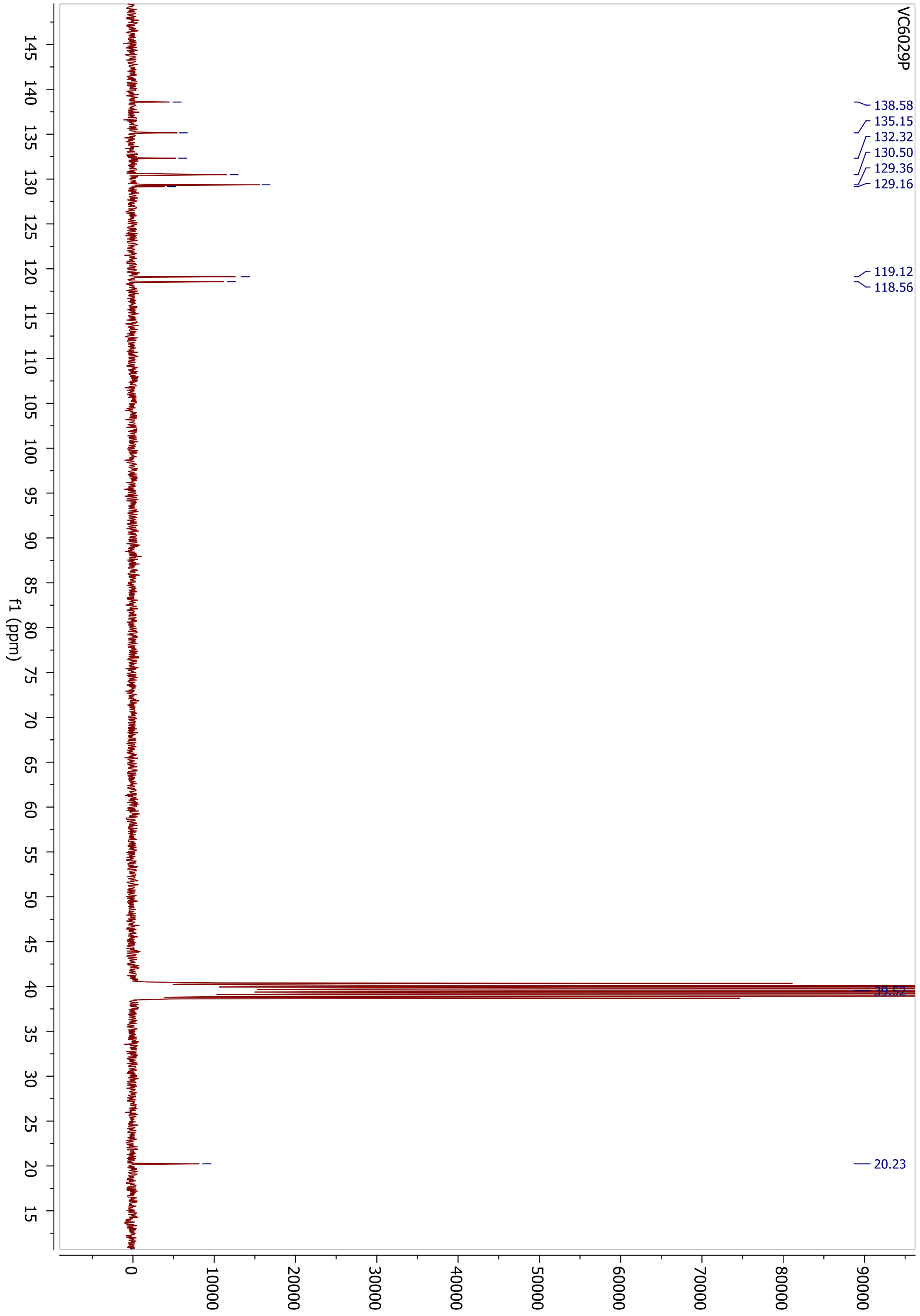

Figure S1: NMR sulfamide **1**.

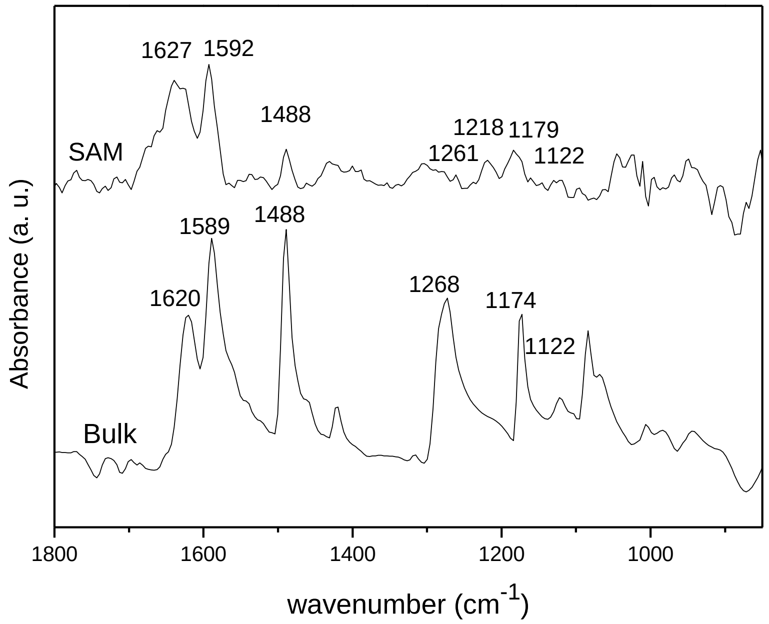

Figure S2: IR spectra of 4-ATP in bulk (solid state) (bottom) and adsorbed on gold (top).

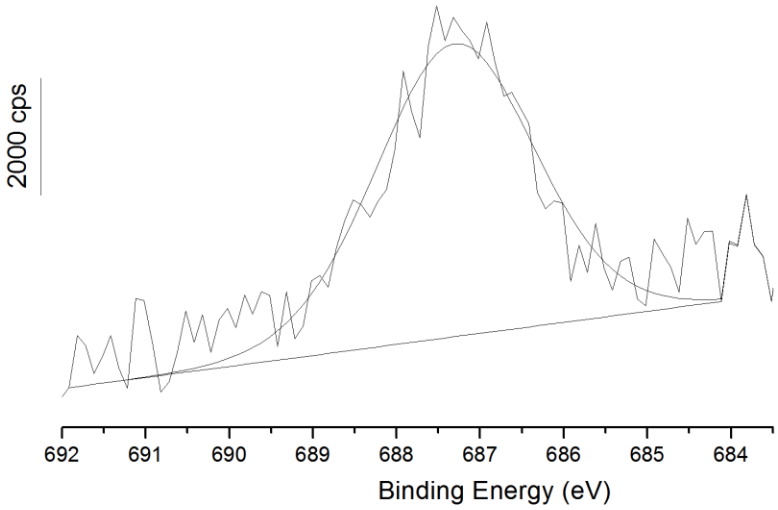

Figure S3: High resolution F1s XPS spectrum of the 4-ATP after reaction with 4-$FC_6H_4SO_2NHOSO_2$-4-FC_6H_4 (SAM b).

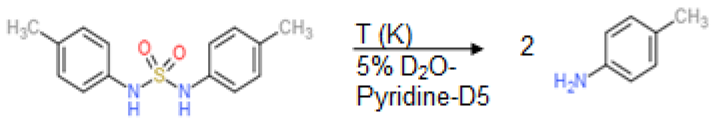

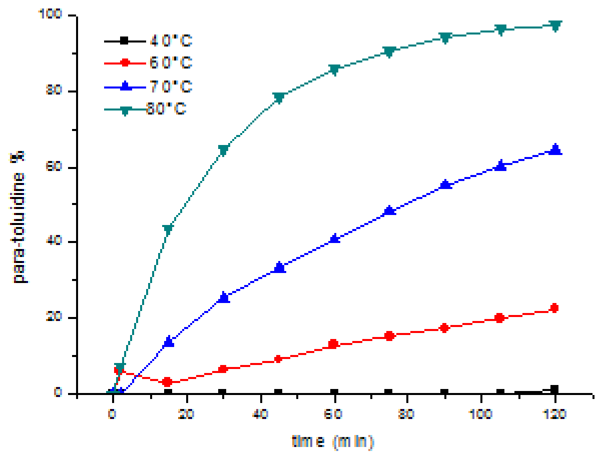

Figure S4: Hydrolysis of a *para*-toluene-derived sulfamide. % formation of *para*-toluidine with time in a NMR tube with deuterium oxide and pyridine-d_5 for different reaction times at one given temperature (40, 60, 70 and 80 °C).
